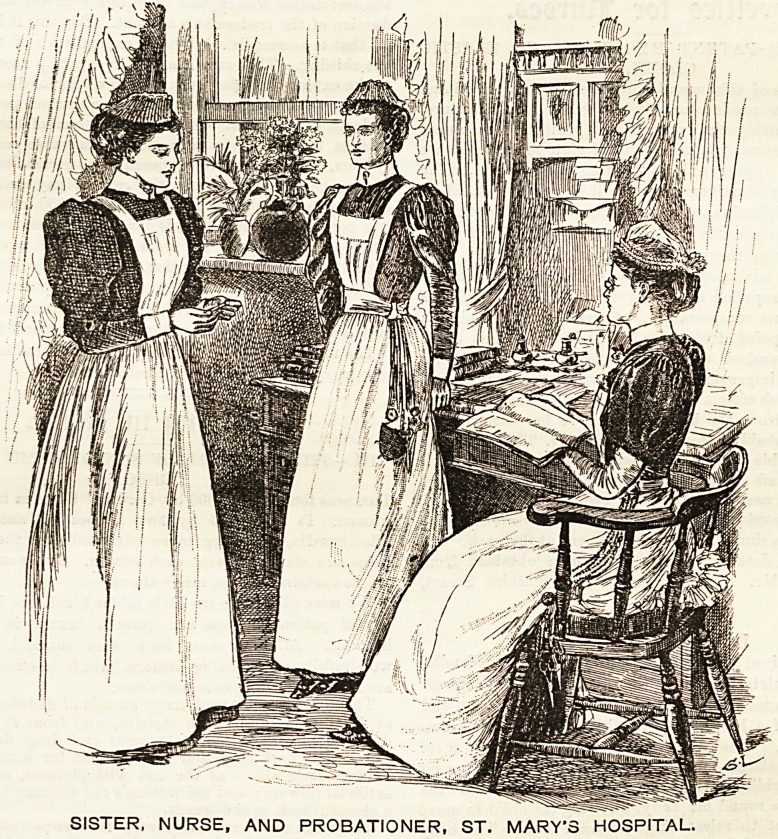# The Hospital Nursing Supplement

**Published:** 1895-10-05

**Authors:** 


"The Hospital" Nursing Mirror.

Being the Extra Nursing Supplement of "The Hospital" Newspaper.

[Contributions for this Supplement should be addressed to the Editor, The Hospital, 428, Strand, London, W.C., and should have the word "Nursing" plainly written in left-hand top corner of the envelope.]

### News from the Nursing World.

### NURSES' QUARTERS AND RECREATION.

A nurses' home for Guy's Hospital is still a dream of the future, which can only become a reality when adequate funds are forthcoming. In the meanwhile, the nurses make the best of their cubicles, which are light and airy in spite of their plainness. No one will rejoice more than the heads of the nursing department when the erection of a proper home is found feasible. All probationers at Guy's Hospital are expected to join the Royal National Pension Fund, and they have substantial encouragement to do so; but present recreation is not lost sight of whilst provision for the future is assured. The night nurses, for instance, have this summer spent three mornings a week in the fine air at Honor Oak, where the hospital tennis grounds are situated, various thoughtful arrangements being made for their comfort and refreshment. When sitting out of doors is no longer feasible, a nurses' choral society will furnish fresh interest for the workers. The matron has kindly offered the loan of her large drawing-room one evening a week through the winter for the practises, and the project has been cordially received. The appointment of an excellent conductor points to a successful development of the scheme.

### HOSPITAL FOR CHILDREN AND WOMEN.

The little hospital for children and women in Waterloo Bridge Road has been undergoing cleaning, repairs, and alterations. The tiling of walls and floors of the lavatories is a satisfactory part of the sanitary improvements. The women's wards, with windows at either end, are fresh and bright, and the patients look contented and comfortable. Two private houses in the neighbourhood have been recently acquired for the nurses' use, and contain a sitting-room and bed-rooms for day and night nurses, the latter occupying all the upper rooms. The present appear to be a great improvement on the former nurses' quarters.

### SUCCESS AND FAILURE.

Attention has been called to the pleasant side of a nurse's life in the columns of the *Daily News* by some admirable letters from nurses of experience. These epistles form agreeable contrasts to those of the ex-probationers, three months' failures, and perplexed parents, who are so fond of talking of their personal grievances. The nurses write out of the abundance of knowledge accumulated during many years of work. "We who know the subject practically laugh at the term 'white slaves,'" writes one hospital nurse, "we find the busy, healthy, happy life of a nurse anything but slavery. Really matrons and committees are not hard-hearted monsters, bent upon sweating us; we find them good friends, sympathising and helpful. We do not wish to be considered before the patients." This writer seems unlikely to put anything or anybody before her duty to the patients, and we are glad to find that in making their welfare her first consideration she herself enjoys a busy, healthy, happy life. Such a type of nurse could hardly be evolved from the young lady who writes that as her payment of a guinea a week to the hospital more than covers the cost of her maintenance, she thinks that instruction in massage, sick cookery, bandaging, and many other departments of nursing, should be included in the privileges of the paying probationer. Doubtless, they would be if she stayed long enough to learn them, but this would take her more than three months. It would be well for a discontented paying probationer to try the experiment of finding board, lodging, attendance, coals, and lights for herself elsewhere, and she would then find out whether she could save out of twenty-one shillings a week sufficient to pay for a course of sick cookery, massage, &c.

### THE PROBATIONERS' PRIZES.

The prize-giving at the London Hospital on October 1st followed the presentation of his portrait to Dr. Hughlings-Jackson, and prizes and scholarships to the students. The fine library at the Medical College was crowded on the occasion, and Sir James Paget, who presided, spoke in high terms of the training of the nurses. Most cordial appreciation was shown by other distinguished speakers of Miss Lückes' admirable organisation.

### NURSES AT LAMBETH.

The large mess-room provided for the nursing staff at Lambeth Infirmary is airy and bright, and the presence of a piano there shows that it has to serve both for recreation and meals. No doubt the Guardians, who have already done so much for the comfort of their nurses, will eventually grant them a study and a sitting-room on the same floor. The bed-rooms are very well furnished and comfortable, and the nurses' sick-room is a most home-like apartment, containing restful easy-chairs and handy tables, besides the other necessary furniture. Even pretty warm shawls, for sitting up in, lie in readiness for invalided nurses. The night nurses' quarters are above those of the day nurses, and there are two night superintendents, who find plenty to do in this very large infirmary. The probationers are lodged in two houses, which have been converted into a very comfortable home. The small single rooms and the cosy sitting-room are furnished with much taste, and "the cottages," as the probationers' houses are generally called, are greatly appreciated. Miss Griffiths (the matron) is to be congratulated on the arrangements for her nursing staff, and on the admirable condition of the infirmary to whose service she has devoted so many years of her life.

### SURGICAL HOME FOR BOYS.

The formal opening of the new home at Banstead took place on St. Matthew's Day, the religious services being conducted by the Rev. E. O. Williams in the presence of a large gathering of ladies and gentlemen, including the chief promoters of the institution. It is intended for the reception of boys suffering from open wounds under thirteen years of age for a payment of 5s. 6d. per week. The difficulty of getting patients of this description admitted into homes is well known to those who have the care of the sick. The house is newly built, large, airy, and bright. It is situated on very high ground (500 ft. above the sea level), and commands a splendid view, and the air is exceedingly bracing. The little patients in the wards are as happy as the most solicitous friends could desire them to be. Applications for admission should be made to the matron. Subscriptions and donations may be sent to R. Wigram, Esq., National and Provincial Bank, E.C.

### LOCAL CRITICISM OF COVENTRY GUARDIANS.

It is satisfactory to learn that the action of the Coventry Guardians respecting the question of night nursing at their Workhouse Infirmary has been condemned by a large number of their fellow citizens. The latter appear entirely to concur with our own view in this matter, and realise that night and day nursing are equally required by the sick. The Coventry Guardians have not apparently grown indifferent to the storm of criticism which they have aroused by their opposition to the institution of night nursing at this infirmary, for the local press reports that much indignation was displayed by them at the recent Board meeting when measures passed by certain ratepayers were brought to their notice. The Coventry branch of the Independent Labour Party passed a resolution supporting the action of those members of the Board who favoured the appointment of a qualified night nurse, and they expressed a hope that their opponents would "recognise that the alleviation of suffering and poverty is of a higher importance than mere shillings and pence." The Coventry branch of the Social Democratic Federation also recorded a resolution passed at a largely-attended meeting, condemning the opposition to a night nurse as "unmanly." The exhibition on the part of ratepayers of so much interest on behalf of the sick is the best possible augury for skilled nursing being in future assured to the destitute poor.

### A NEW SOCIETY.

At a meeting recently held at Alyth Town Hall, it was decided to establish a district nursing association for Alyth and Meigle. Sir James Ramsay, Bart., took the chair at the meeting, and the Dowager Countess of Airlie was appointed president of the society, the vice-presidents being Lady Ramsay, of Banff, and Lady Kinloch, of Kinloch. A very interesting address on sick nursing was given by Miss Guthrie Wright, hon. secretary of the Scottish branch of the Queen's Jubilee Institute.

### TWELVE TO SIXTEEN.

Complaints have reached us on more than one occasion as to the difficulty of finding convalescent homes which admit boys between the ages of twelve and sixteen. One correspondent says he believes there is scarcely a home in the United Kingdom which will take them, and adds, "I am aware that there are difficulties in dealing with lads about this age which do not arise with either younger or older persons, but surely they may be overcome." We shall be glad to hear from more of our readers on this subject, and to learn whether there is really urgent need for further provision for convalescent boys between twelve and sixteen.

### THE INDIAN NURSING SERVICE.

The resignation is announced of Miss Barker, lady superintendent at Poonah. She returns to England in December. Miss Barber was a sister at King's College Hospital, and afterwards head of the Bombay command. A deputy superintendent is also retiring this autumn, so there will be two important vacancies to fill in the Indian Nursing Service.

### OVER-MANAGED.

Why should it be thought necessary to select their recreation and associates for nurses? The fact that most people gladly extend hospitality to nurses when they are off duty appears insufficiently realised. Besides, it is evident that nurses, like every other class of women, have individual interests and personal friends and relations, and many of them begin to feel a little satiated with the prevailing tendency to dictate to them all that they ought to do. Nurses are generally quite self-reliant and intelligent enough to dispose of their own leisure without being told whether they are to be entertained either as "hired servants" or "honoured guests." An article in a rural contemporary contains an amusing little story of a great lady who disappointed an ambitious hostess by staying away from a party to receive her own daughter—a hospital nurse. She explained that she could not venture to bring her, as the hostess had previously refused to entertain other nurses because they were "hired servants."

### AMERICAN TRAINING SCHOOLS.

The designation, "Training School," is applied in America in a much wider sense than in England, being used even when the "graduating class" (namely, probationers who complete their training) numbers only two or three members. That the smallest hospitals adopt the title of "school" for their nursing department is constantly shown through the interesting reports given by our American contemporary, *The Trained Nurse*. Whether or not diplomas granted at small schools are as valuable as those given in larger ones, it is obvious in such a great continent there are many advantages in a wide distribution of hospitals and trained nurses.

### OUR NEEDLEWORK COMPETITION.

Now that we are entering the fourth quarter of the year it is time to remind our readers of our annual needlework competition. We hope that the garments sent in for the Christmas distribution will exceed in number those of other years. We always receive many pretty and useful things for the sick poor who spend Christmas Day in hospital, but the demand exceeds the supply, and we trust that our readers will do their best to enable us to respond to the appeals made for this practical form of help. The prizes offered are: One guinea for the best flannel dressing-gown, 10s. for best bed jacket (for man or woman), 10s. for best flannel shirt, 5s. for best flannel petticoat, 5s. for best over-petticoat, 5s. for best knitted socks, 2s. 6d. for second best pair, 2s. 6d. for best warm vest (for man or woman). We hope that the suggestion made in another column by a nurse now in Paris will commend itself to others. If those who are willing to provide materials, but have no time to make them up, would allow others with more time than money to undertake the needlework, the sick poor would greatly benefit. If this idea meets the wishes of our readers, we shall be pleased to put those who will write to us on the subject into communication with each other.

### Elementary Physiology for Nurses.

By C. F. MARSHALL, late Surgical Registrar Hospital for Sick Children, Great Ormond Street.

### IX.—THE RESPIRATORY SYSTEM (*Concluded*).

### Changes in the Blood Caused by Respiration.

We have seen that the blood is not alike in all parts of the body. We have seen the broad distinction between arterial blood and venous blood, the former being richer in oxygen and food, the latter containing more carbonic acid. There are further differences of great importance in various parts and at different times. As the nature of the water in a river will depend at any place on the supply of pure water by streams, on the animals dwelling in it, and on various matters received from the banks and discharged into it by drain-pipes, so it is with the blood. If we compare the stream returning from a muscle at rest or in activity, from the stomach during digestion, and in the interval between meals. Blood from muscle, brain, and stomach can never be quite alike, as these organs abstract different matters from it.

Let us confine ourselves to the changes effected in the lungs. Venous blood from the right auricle is of a dark purple colour; arterial blood from the left auricle is bright red. This change is effected in the lungs, and depends on gases, for if we shake up venous blood with air or oxygen it becomes arterial, and if we shake up arterial blood with carbonic acid it becomes venous after a time. The amount of gases in the two cases is shown thus:

| 100 Volumes of blood yield ... | O | $Co_2$ | N |
|---|---|---|---|
| 1. Arterial blood ... .. | 20 | 39 | 1·2 |
| 2. Venous blood ... ... | 8-12 | 46 | 1·2 |

It will be seen that arterial blood contains twice as much carbonic acid as oxygen, while venous blood has five times as much.

### Relation of Oxygen to the Blood.

The oxygen is not simply dissolved in the blood, but is in chemical combination with the hæmoglobin of the red blood corpuscles, which has a great affinity for it. The hæmoglobin gives up oxygen to bodies having a stronger affinity for it, *i.e.*, to the tissues of the body, and goes back to the lungs for a fresh supply. Poisoning by carbonic acid is due to the fact that hæmoglobin has a greater affinity for carbonic acid than for oxygen, and hence combines with it by preference, thereby causing death from absence of oxygen.

### Relation of Carbonic Acid to Blood.

The carbonic acid is contained in the serum, not in the corpuscles, and is probably in part dissolved, but in part in loose chemical combination, possibly as bicarbonate of soda.

### Changes in the Tissues.

The tissues are the real seat of manufacture of carbonic acid; and other waste products are formed, as we considered in the second lecture. The temperature of the body is maintained by the chemical processes going on in it, for heat is set free whenever chemical changes occur. The regulation of the bodily temperature is under the control of the nervous system. This power of controlling the production of heat is shown by the fact that temporary application of moderate cold raises the bodily temperature, and *vice versâ*; and that cooling of the external surroundings increases the production of carbonic acid and the consumption of oxygen, and also increases the temperature. The temperature is further regulated by means of the skin. Heat causes the cutaneous vessels to dilate, and also causes an increase of sweat by acting on the nerves governing the production of sweat. Now, the more blood that passes through the skin the greater will be the loss of heat by radiation; and the greater the amount of sweat, the greater will be the loss of heat due to its evaporation. Cold, on the other hand, contracts the vessels of the skin and diminishes the amount of sweat, thus diminishing the loss of heat from the skin. The skin is thus the great means of regulating the loss of heat from the body. The nervous mechanism regulating the production of heat is more complicated, and not so completely understood. It will suffice to state that the production of heat is supposed to be under the influence of a special part of the brain.

### Excretory Organs.

The importance of these we have already considered. We saw that the body was perpetually manufacturing poisonous bodies within itself, and that the duty of the excretory organs was to get rid of these bodies. We also saw that these products were formed as a result of oxidation, whereby more complex bodies were split up into simpler ones. Of these bodies carbonic acid is the most important, and this is excreted by the lungs, which are excretory as well as respiratory organs.

The kidneys are the chief excretory organs, and they get rid of the next most important waste product—urea. The shape of the kidneys is well known, and when examined under the microscope they are seen to consist of a mass of small tubes, surrounded by loops of blood-vessels. The cells forming the walls of these tubes separate the urea from the blood circulating around them. The tubes ultimately open into a receptacle in the centre of the kidney, which leads to the ureter, and by this the excretory matter in the form of urine is conveyed to the bladder, and so got rid of. Water is got rid of also by the kidneys, forming the bulk of the urine. The lungs and skin also excrete water, and the amount of water excreted by the kidneys varies with that got rid of by the skin. Hence the diminished amount of urine passed in hot weather and the increased amount in cold weather.

### Nursing in Ireland.

The provision of adequate night nursing for the sick poor in Irish workhouses will receive an impetus from the recent action of the Local Government Board for Ireland. The step that has been taken is sufficien ly important for us to give the official communication at length. The power of the Local Government Board is more circumscribed in England, and their recommendations respecting trained night nurses cannot be enforced in the same way on this side the Irish Channel.

Local Government Board, Dublin,
September 20th, 1895.

Sir—The Local Government Board for Ireland acknowledge the receipt of the resolutions adopted by the Board of Guardians of Athlone Union on 7th inst. declining to appoint a night nurse in the infirmary, and the Board desire to state that in adopting this resolution the Guardians have disregarded the recommendations of their medical officer, the censures passed by the coroner's juries upon the arrangements for night nursing, and the facts disclosed at the inquiry, on oath, which led to the recent correspondence on the subject.

The Local Government Board, throughout the correspondence which has taken place, have spared no efforts to awaken the Guardians to a sense of their obligations towards the sick poor, in connection with night nursing in the workhouse hospital; and in view of the final refusal of the Guardians to appoint a trained nurse for night duty, the Local Government Board are compelled reluctantly to come to the conclusion that, in the interests of the sick poor, it is now their duty to place the management of the union in other hands, and they accordingly enclose an order, under seal, dissolving the Board of Guardians of the Athlone Union.

D. J. M'Sheahan, Assistant Secretary.

To the Clerk, Athlone Union.

### Neuralgia and its Treatment.

By Rowland Humphreys, M.R.C.S., L.R.C.P., &c.

The term neuralgia means nerve-ache, and consequently is merely the name applied to a symptom. The treatment of neuralgia, to the public mind, means the relief of the ache, and entirely disregards the cause to which the pain may be attributed. In medical parlance it is said that neuralgia is the expression of malnutrition of the nerve supplying the area where the pain is felt. Thus it is easy to understand that the popular use of antipyrin, antifebrin, phenacetine, and other drugs which relieve pain, and which may be obtained at any chemist's without a medical man's prescription, is attended with a very serious danger. Pain is one of the great "storm signals" of nature. It warns us that something is wrong, and by applying anatomical knowledge it is usually tolerably easy to hit upon the cause. Pain, inflammation of a nerve, pressure upon it at any point of its course, irritation of any of its branches is felt at the termination of the nerve in skin, bone, or other structure, and if the cause be sufficiently severe or sufficiently prolonged the pain is "reflected," or extended along nerves closely connected with the one immediately affected to, perhaps, distant parts of the body. Thus the pain of an inflamed hernia is often conveyed to the pit of the stomach, that pain due to pleurisy may be felt in the stomach, and that due to irritation in the rectum may be transmitted to the heel. If, therefore, an ignorant person, having an ache of some description, walks into a chemist's shop, and, asking for something for the pain, obtains a drug which relieves the pain without removing the cause, he may very well get into great trouble, because he has hauled down the storm signal before his ships were safely in harbour.

Every ache is a neuralgia, but the term is generally applied only to a pain occurring in the course of some nerve, for which we are unable to find any local definite cause. If any definite cause can be assigned then the name of the organ or structure which is disordered is prefixed to that of the word neuralgia, and by the collective term we mean that there is some condition of disordered nutrition which has not gone so far as to produce disease.

The general causes of neuralgia, are, in the first places, a predisposition in the patient's family to the development of various nerve affections, such as hysteria or epilepsy. If with this predisposition an undue strain in any direction is superadded, especially if it be long-continued neuralgia, is pretty certain to appear, and, if its warning be unheeded, the graver forms of nerve disease, such as those already mentioned, are likely to make their appearance. Thus, in a recent case, the patient had suffered from severe headaches which had lately increased so as to become continuous; being at school they were neglected until an attack of epilepsy supervened; it was then found that the patient was suffering from defective sight, and that this was putting a severe strain upon his nervous system, and accounted for the headaches and for the fits. He came of a nervous family.

Anything which depresses the general health will make itself shown in the part of the body which is the most subject to strain, or which is badly nourished. Thus it is that neuralgia is most often seen in anæmic, badly nourished persons, and, since they nearly always have bad teeth, the nerves which supply the jaws, or someone of their branches or connections, is where the pain is situated. Thus it may either appear along the lower or upper jaw, or may shoot up into the head, or into the temple, or even appear at one of these spots only, and they may lead to a mistaken impression as to its cause. Where some defect in the eyes is the cause of the neuralgia the pain is situated either over them, or at the back of the head, or may pass down the spine.

Pain travelling up the back of the neck to the head may also be due to the teeth, but is often a form of spinal neuralgia derived from general debility.

Pain travelling along the course of the ribs, that is affecting the intercostal nerves, is frequently left after an attack of "influenza," or herpes zoster, and the tendency remains for a period of months after the attack. The intercostal nerves are also the seat of pain in pleurisy. The lower intercostal nerves, in the same way, are the seat of the pain in peritonitis, and in other abdominal affections.

Spinal neuralgia, a very troublesome form of the complaint, is a neuralgia proceeding from conditions of general weakness, such as neurasthenia; in it the pain is virtually a form of intercostal neuralgia, but is generally situated along the course of the spine, appearing less often in the side or front of the chest. The intercostal nerves spring from the spinal cord some two inches above the rib they run along, and have three branches, one near the spine, one in the side, and one in the front of the chest; in intercostal and spinal neuralgia pain is sure to be felt in all these situations at one time or another.

Sciatica is another form of neuralgia. It affects the sciatic nerve, the great nerve which supplies the greater part of the muscles of the lower limb; in it the condition not uncommonly passes beyond a mere disorder of nutrition, and becomes one of disease, and signs of severe nerve injury may appear, the nutrition of the limb suffering, muscles wasting, the skin becoming glossy, and hypersensitive or the reverse, and the limb becoming weak.

Besides the terminations of the nerves, there are other points where pain is felt in neuralgia. These points, situated at the place where the nerve issues from a bone, are known as the points of Valleix. One such point is situated near the junction of the middle and inner third of the upper margin of the orbit.

The pain in sciatica is often felt in the nerve near where it issues from the pelvis, or lower down along the course of the nerve, or of its branches, and especially behind the upper end of the fibula. Neuralgias of the intestines, or of the stomach, are very troublesome affections to treat, and there is always a danger that the pain of gastric ulcer may be mistaken for that of neuralgia. The chief points characteristic of neuralgia are that the pain comes on before food, and that although it may be increased by it for a short time, it soon passes off. The pain in gastralgia is, too, more diffused than in gastric ulcer, and there is commonly an occasional vomiting of blood or "coffee-ground" material. The symptoms of cancer of the stomach are very similar to those of ulcer.

Sometimes neuralgia remains as the result of old inflammation, as after peritonitis, or inflammation affecting any other part of the body.

(*To be continued.*)

### Notes and Queries.

### Queries.

(1) *Massage.*—Where can I get information as to an association of reliable masseuses, certificates, &c.?—*Nurse B.*

(2) *Books.*—Where can I get the last edition of Honnor Morten's "How to Become a Nurse"?—*E. M. T.*

(3) *Lip-reading.*—Can you tell me of a manual on lip-reading for a deaf person.—*M.J.M.*

### Answers.

(1) *Massage* (*Nurse B*).—You had better write to the Hon. Secretary of the Society of Trained Masseuses, 12, Buckingham Street, Strand, for advice on the subject of which you write.

(2) *Books* (*E. M. T.*)—Write to the publishers, Scientific Press, 428, Strand.

(3) *Lip-reading* (*M. J. M.*)—You had better write to Mr. Van Praagh, Association for Oral Instruction of Deaf and Dumb, 11, Fitzroy Square, London.

### Dress and Uniforms.

By a Matron and Superintendent of Nurses.

### ST. MARY'S HOSPITAL.

Navy blue cashmere made plain and full is the garb which characterises the sisters of St. Mary's Hospital. Coat-shaped sleeves fit comfortably to the arm, allowing that freedom of action so essential to the efficient performance of those varied duties which not infrequently fall to the lot of those who are in charge of a sick ward. Fine linen cuffs relieve the monotony at the wrists, and a collar of becoming width makes a finish at the neck. A linen apron of ample dimensions is worn over the dress, and is provided with a square bib which fastens on to the bodice in front. Pretty light-looking spotted net forms the material for the cap, which is round in shape, fitting neatly to the head, and edged with a quilling round the front that ingeniously develops into a ruche at the back with excellent effect.

The staff nurse wears a dark grey homespun, made quite plain. The skirt is full and turned up with a hem that just clears the ground allround. The bodice buttons down the front, and is fixed into a band at the waist, to which the skirt is attached. The white linen apron is completed with a bib and straps that cross behind, fastening on to a button at the waist. Not unlike the sisters' is the cap of spotted net. The shape differs slightly, in the case of the nurses being rounder and flatter. Two rows of goffered net with a thread run through them draw the cap into the required shape. A tiny blue and white checked galatea dress marks the probationer. This, like those already described, is made quite plain and off the ground. The other details of the costume—apron, cuffs, collar, and cap—correspond exactly with those worn by the staff nurse, and appear to be well made and carefully and neatly adjusted.

SISTER, NURSE, AND PROBATIONER, ST. MARY'S HOSPITAL.

### DRESS NOVELTIES.

It is always a pleasure at the commencement of a season—be it spring or autumn—to receive the comprehensive and well-assorted patterns of woollens and serges produced by Mr. Egerton Burnett, Royal Serge Warehouse, Wellington, Somerset. There is constantly something new, something pretty, and, moreover, something useful in the selection, and that person would be difficult indeed to please who did not experience covetous as well as pleasurable sensations at the sight of so many attractive novelties. The most popular, perhaps, is the famous "Royal Serge," which becomes more of a favourite with the public every day. It is admirably adapted alike for the yacht, the moor, and the hospital, and will be found equally suitable for wear under these three somewhat dissimilar conditions. Homespuns also are offered in great variety, and at most reasonable prices. To these must be added a long array of zephyrs and galateas, suitable for nurses' uniforms. The zephyrs are particularly light and fine in texture, and there are some charming silver greys that would be found delightful for wear in the sick-room.

The striped galateas, appropriately termed the "Nightingale Stripe," are now offered in three colours, namely, green and white, blue and white, and black and white. The former is very uncommon, green as yet being a colour little patronised by the nursing profession. The stripes are so narrow that all hardness of outline is obviated, and the white has a softening effect that would be very becoming to the wearer. Mr. Burnett has also a large tailoring and dressmaking department, where, at a very moderate charge, materials on selection can be made up in any style that may be desired—a very great convenience to the majority of our readers, who have little inclination and less time for making up their own costumes.

### Novelties for Nurses.

### ICKRINGILL'S PATENT HYGIENIC AND ELASTIC CLOTH.

The inventors of this useful cloth are to be congratulated on their efforts to produce an article which is light in texture, elastic, yet affording the warmth and protection necessary to the body. From a hygienic point of view the samples we have before us appear to leave nothing to be desired. The nature of this cloth eminently adapts it for elastic stockings and bandages, abdominal belts, and other surgical appliances where firm even pressure is desired. It lends itself with great readiness to the varied positions of the body, thus possessing many advantages over the ordinary articles in use. It can also be supplied at a very much less cost, which is another important consideration. A great recommendation from a sanitary point of view lies in the fact that this cloth can be easily washed without in any way impairing its quality, a great improvement on the ordinary surgical belt or stocking, which after a time becomes unpleasantly redolent of perspiration from the body, and has to be discarded on that account probably before being worn out. The abdominal belts are admirable in style and shape, and are not only well cut, but contrived in such a manner as to insure support where it is most needed. They are beautifully finished off, and can be adjusted without the usual arrangement of straps and buckles by a simple lacing arrangement at the back. An ample catalogue detailing prices, &c., can be obtained from the secretary, Mr. E. J. Pryse, 35, Devonshire Street, Keighley.

### BABY FASTENER.

We have received from Messrs. Wilson and Co. (of Kendal) an ingenious contrivance which they term a Baby Fastener. This is an appliance for restraining infants in their cots, and it is composed of a broad unshrinkable web band with two overlying flaps. The web band is placed upon the sheet covering the mattress, and secured beneath the latter by strings. The child is then laid upon the band, and the two flaps outline round his body, allowing the child to move at will, but not to release himself from them. The baby fastener is made of washable materials, and the brass stays at the end are made removable for that reason. We would, however, suggest that, by way of improvement, it might be better to construct the baby fastener of a rather softer material, as we think that which is at present employed too stiff and hard.

### A NEW INSURANCE.

Safeguards against loss of life and property, of one kind or another, are sprung upon us from all sides nowadays. The Scottish National Key Registry and Assurance Association, first instituted on a very small scale in Edinburgh some ten years ago, is a rather quaint outcome of the growing desire on the part of the general public to be beforehand with every kind of minor misfortune, with compensation for loss on a larger scale thrown in as it were.

"Much worry and inconvenience," says the circular of the association, "is caused by the loss of one's keys, and the delay and expense of advertising for them are often considerable." To ensure against this worry and inconvenience the association undertake to supply, for the nominal sum of one shilling per annum, a German silver label, having on one side a distinctive number (which is allotted to the subscriber in the association's registers), and on the other side an inscription requesting the finder of the keys, in the event of their being lost, to take them with all speed to the local police office, for which he will receive a reward of 5s. At the police stations copies of the register are kept for the use of officials only, the finder remaining thus in ignorance of their ownership. Should the label fail to bring back lost keys, the association will replace them to the extent of £5 on production of the tradesman's account. But this is by no means all that is accomplished by the expenditure of the original one shilling. That same small coin covers personal accident to the extent of £1 per week for ten weeks for disablement, or £100 to the legal representative of an assured person in case of death by accident. For the use of one of the key labels entitles the holder to an accident assurance coupon. For cyclists, whose risks are supposed to be greater than those of the less enterprising people, the rate of payment is £1 per week for five weeks for disablement, and £50 in case of death. One penny, we should add, is charged for the coupon cover, which contains space for memoranda, and several useful little bits of postal information.

The head offices are at St. Andrew's Square, Edinburgh, and the London office, whence all information may be obtained, is 3, Broad Street Buildings, Liverpool Street, E.C.

The idea has been adopted by various offices in the South, who carry it out on much the same principle.

### Nursing in Rome.

### III.—THE HOSPITAL OF SAN GIACOMO ON THE CORSO.

This was founded in 1300 by Cardinal Colonna for men and women. It is served by two classes of sisters of the Misericordia, one being more educated than the other, and there are six doctors to each section. The women's wards are downstairs and the men's above.

We were pleased to see little tables beside the beds for the use of patients; these are quite a luxury in an Italian hospital. All the cases here were surgical, and some wonderfully successful operations have been effected. There are four rooms for bandaging alone.

The theatre contained fixed barrels of disinfectants, such as we had seen in Santo Spirito, and from it by a glass door we pass into the instrument and drug departments. There were both large and small wards for women, several having little altars at the end with pictures, candles, and artificial flowers; and the women's red dressing jackets gave a cheerful look to the wards.

The store-rooms and dispensary for out-patients are quite separate. There is a large laundry on the premises. At the time of our visit workmen were busy in the garden building a new deposit ward, so that accidents could be brought straight into it from the Corso. The whole building was undergoing cleaning and repairing, so that we got but an imperfect view of it, but what we saw was very satisfactory. We went through it with Dr. Raffaello Bastianelli.

The interest of the King of Italy in hospitals and in the poor was shown a week or two before our visit, when a house fell down in one of the streets of Rome, and buried a working man in its ruins. News of the accident was brought to the King while he was at breakfast. Without a moment's delay he left his meal and hastened to the spot. It was some hours before the unfortunate man could be extricated, and from eleven in the forenoon till four in the afternoon the King remained by him, holding his hand and consoling him. At length, when the work of rescue was completed, the King walked by the litter to the hospital, and visited the man constantly while he continued a patient.

### French Schools for Trained Nurses: Their Origin and Organisation.

By Madame W. Vignal.

### VI.—PARIS HOSPITAL NURSING STAFF.

From 1890 91, 135 pupils were candidates in the practical examinations who belonged to the following categories of pupils: With scholarships, 7; male nurses from the Pitié Hospital, 14; female nurses, 25. Male nurses from other hospitals, 28; female nurses, 45 Male pupils not attached to any hospital, 2; female pupils, 14.

From 1891-92, 190 were candidates in the practical examinations: With scholarships, 6. Female nurses from the Pitié Hospital, 30; male nurses, 19. Male nurses from other hospitals, 69; male nurses, 47. Unattached pupils, men, 15; female, 4.

From 1893-94, 151 pupils were candidates at the practical examinations: With scholarships, 5. Female nurses from the Pitié Hospital, 30; male nurses, 12. Female nurses from other hospitals, 48; male nurses, 33. Unattached pupils, 20; male, 3. These statistics show that on an average the female pupils are more numerous than the male pupils at the classes and likewise at the examinations.

The practical classes are over looked by a *surveillante* (female superintendent). Dressing and bandaging are taught, and also how to give baths of all kinds, douches, &c., and how to dry-cup by means of placing bell-shaped glasses, called "ventouses," on the affected region. During the last few years practical lessons how to dress new-born infants have been given. There is not a maternity service at the Pitié Hospital, where, whenever a confinement takes place, the opportunity for this form of practical lesson is utilised. Recent years have also brought about another important improvement in the practical lessons given at the Pitié Hospital; they now form part of a corporate whole, inasmuch that in four other hospitals similar series are organised, each filling up a hiatus left by the other.

At the Lariboisière Hospital the classes are directed by a *surveillante diplomée* (certificated superintendent). They are held three times a week from October until the following July. The pupils attending them belong to the nursing staff of the Lariboisière Hospital, of the *Maison de Santé*, of the St. Louis Hospital, and of the Bichat Hospital. The number of pupils in October, 1893, was 63; in July, 1894, 38, including four unattached pupils.

At the Cochin Hospital the classes are directed by a *suppleante diplomée* (certified superintendent). They were opened on November 16th, 1893, and terminated on July 19th, 1895, were held on 146 occasions and were regularly attended by 34 pupils.

At the Necker Hospital the classes were attended by four of its male nurses and six of its female nurses, 14 female nurses from the Children's Hospital, and two pupils from without. The classes terminated in June. The numbers are unsatisfactory, and it is to be hoped that succeeding years will see an increase.

At the Tenon Hospital a *surveillante diplomée* (certificated superintendent) also directs the classes. They begin in the first fortnight of December, and are held three times a week from four to six o'clock p.m. The course from 1893 to 1894 was regularly attended by 12 pupils. At the Necker and the Tenon hospitals the number of the nursing staff attending the classes is deplorably small.

The practical classes of the four above-named hospitals furnish the same kind of teaching as that given in the Salpetrière, Bicêtre, and Pitié hospital schools. It is requisite that all these classes should begin in November, that they should be regularly given on the days and hours previously decided on and continue until the July examinations of the following year. It is of the greatest importance that they should be attended by the pupils of the Salpetrière, Bicêtre, and Pitié schools in order that the instruction afforded by them should form a complement to that provided by the three parent schools. The Pitié gives the greatest number of diplomas each year, and its classes are attended by a larger number of pupils than those of the other hospitals.

From 1891-92, 163 diplomas were granted to the following candidates: *Sous-surveillants* (male sub-superintendents), 2; *sous-surveillantes* (female superintendents), 7; *suppleants* (head male nurses), 4; *suppleantes* (head female nurses), 10; scholarship pupils, 6; male nurses, 43; female nurses, 74; unattached pupils, 17; total, 163.

From 1892 to 1893, 143 diplomas were given as follows: *Sous surveillante* (female sub superintendent), 1; *suppleant* (head male nurse), 1; *suppleante* (head female nurse), 1; scholarship pupils, 5; male nurses, 58; female nurses, 60; unattached pupils, 17; total, 143.

From 1893 to 1894, 135 diplomas were granted to the following pupils: Head female nurse, 1; scholarship pupils, 5; first or head male nurse, 1; male nurses, 41; first or head female nurse, 1; female nurses, 62; unattached pupils, 34 total, 135.

The above statistics show that of the three Paris nursing schools, the school of the Pitiê Hospital confers the greatest number of diplomas. If the average is taken the result is as follows: The yearly average of the Salpetrière School is 82, of the Bicêtre School 77, and of the Pitié 135. From 1883 to 1894 the schools for male and female nurses have granted 1,755 diplomas to candidates of both sexes, the nominations of 1893 and 1894 included. These schools have always provided the Paris hospitals with a large proportion of their subordinate nursing staff, who are undoubtedly superior and more capable than they formerly were, when the classes entered on their duties thoroughly ignorant of their duties, and it is easy to foresee that in a very short time both male and female nurses under forty years of age will have a good knowledge of their professional duties.

It is now some time since Dr. Bourneville conceived and carried out the idea of organising a museum containing every necessary for dressing, ordinary drugs, instruments, and surgical appliances. At the Bicêtre Hospital, with the co-operation of a patient, he has made a collection of designs representing all the different kinds of dressings. Last year, aided by some zealous assistants, Dr. Bourneville supplied the want he so greatly deplored at the Pitié Hospital, where the Scholastic Professional Museum has by slow development become an excellent collection of the kind.

The lectures at the three schools are given free, are open to all hospital nurses without distinction, to private nurses and mothers of families, and in fact, to the public generally. Dr. Bourneville is desirous of effecting a change in the economic organisation, and his reports to the Municipal Council have dealt with the shortcomings of what may be called the domestic comforts allowed to the Paris hospital nursing staff. His addresses given on the occasion of the distribution of prizes of his hospital schools also refer to this subject. Dr. Bourneville wishes that the general dormitory should be converted into separate bed-rooms, and furnished with regard to the necessities of cleanliness. A common dormitory brings about deplorable results. The doctor has obtained some concessions to his views at the Pitié Hospital, which greatly needs improvement, and where next to nothing has been done in the direction indicated. Both male and female nurses are badly lodged.

### Holidays and Health.

[Readers of The Hospital in need of information about health resorts at home or abroad, or desirous of aid in forming holiday plans, are invited to send queries to Editor, 428, Strand, W.C. (marked "Travel" on outside of envelope), which will be answered under this section.]

### IRELAND.

All sorts of efforts are being continually made to induce the English tourist to prefer a sojourn in the sister isle to the regulation trip to the Continent. But the English tourist remains obdurate. When he has once brought himself to the point of carrying himself and his purse across a strip of stormy water, it is the English, not the Irish Channel which he selects for the scene of his miseries. He is indifferent about developing British trade, and as for the sound of his own language, which he is assured will be a solace to him in a strange place, he would rather get beyond it, and be cheated if needs must under the disguise of an unfamiliar coinage. The fact is neither argument nor persuasion is of much avail against the indefinable common impulse which regulates where we shall go and what we shall do. Ireland has only to wait, to set her house in order and spread her net for the visitor. Some day, perhaps very soon, for signs are setting in that direction, the little imperceptible breath of fashion will veer round westward; Erin will be "discovered" and annexed to the territory of the tourist, and, as the force of her charms prevail, the associations, which in the most peaceful households have made her name a watchword of strife, will fade away into oblivion.

What has she to offer? To the tourist pretty nearly everything that this ubiquitous individual is accustomed to expect when he makes his annual emigration. Mountains, lakes, caverns, waterfalls, islands, grand rocky coasts, level sandy shores, an ever-varied succession of beauties, the romance of which is familiar to everyone in the clinging melodies of Moore, but whose details are as little known to the general public as the sands of Araby. Moreover, the tourist need forego none of his usual comforts. In all the better known places of interest he will find decent accommodation, and in many of them really good hotels. It goes without saying that he will find good humour and civility wherever he goes to make up for all deficiencies, and that is no small ingredient in the enjoyment of a holiday. A network of railways, supplemented by an excellent service of coaches, extends now to all parts of the island, and in less frequented routes the "low-backed car" is still to be hired, and at a reasonable rate. It is hardly superfluous to speak of these details, for it is very common to hear people speak of Ireland as though it had gained little in civilisation since the days of Elizabeth; and many who would not care to admit the feeling, labour under an impression that they may chance to serve as a mark for the "moonlighter," taking "pot-shots" at his landlord.

For the invalid Ireland offers advantages of climate fully equal to those of many far-famed Continental resorts. A useful handbook by Dr. Flinn* considers the country from this standpoint, and affords a great many statistics to show what places should be selected as health resorts in summer and winter. The great point for the invalid is the equable temperature. Ireland is alike free from extremes of heat and cold, and is as well suited to those who dread the glare and oppression of a hot summer as to those who wish to escape the rigours of winter. For those consumptives for whom a warm, moist atmosphere is indicated few places afford more advantages than Glengariff, charmingly situated at the extremity of the gulf which runs inland from Bantry. On the north, east, and west it is surrounded by lofty ranges of mountains, and is thus entirely protected from cold winds. It is entirely free from fog, boasts more than its share of sunshine, and has a mean winter temperature of 45 deg. Many patients, too, suffering from bronchial affections and asthma, have experienced great benefit from a residence at Glengariff. The journey, always an important consideration with invalids, is comparatively easy. From Dublin it can be reached in ten hours, including the drive from Bantry, —which ranks as one of the most beautiful in the country— and a good service of trains connects Bantry with Cork at the distance of about an hour. Concerning the charms of Glengariff the best comment is Thackeray's exclamation, quoted by Dr. Flinn: "What sends picturesque tourists to the Rhine and Saxon Switzerland, when at Glengariff there is a country the magnificence of which no pen can give an idea? Were such a bay lying on English shores it would be a world's wonder."

A great effort is being made to revive the fame of several Irish mineral springs which at one time enjoyed a good repute. The only warm spring known in Ireland is at Mallow, where the water issues at a temperature of from 70 to 72 deg., and the consequences of imbibing these watert inadvisedly have proved so serious that the experimens might well be declined even under proper safeguards.

The cold spa at Lucan, within a few minutes by rail and tram of Dublin, was famous at a time when Buxton, Harrogate, and Tunbridge Wells were scarcely thought of, and has of late years regained a large share of its former popularity. "The action of the water is stimulant, mildly alterative, and slightly diaphoretic, and is especially beneficial in chronic cases in the various forms of skin disease."

Lisdoonwarna combines the advantages of a mineral spa and a bracing seaside resort; it is situated within three miles of the Atlantic, at a distance of eight hours by rail from Dublin, and the vicinity of a large tract of bogland is quoted as one of its advantages. The famous Gowlaun sulphur spring is the one chiefly resorted to, and its waters are found principally efficacious in cases of skin disease, long-standing rheumatism and gout, chronic congestion of the liver, and scrofulous affections of the bones and joints. In former days it was usual to export the water, which was drunk in large quantities in England for the benefit of the complexion. Readers of "Persuasion" will remember that Sir Walter Elliott attributed the cure of Mrs. Eley's freckles to "the use of Gowlaun, nothing but Gowlaun," persevered with for a lengthened period, and could hardly be persuaded that the improvement in Anne's looks was not due to the same cause.

* "Ireland: Its Health Resorts and Watering Places." G. D. Edgar Flinn, F.R.C.S., D.P.H. With numerous illustrations and maps. (London: Baillière, Tindall and Co.)

### Where to Go.

People's Concert Society (Season 1895-96).—1. The Town Hall, Poplar, E., at eight p.m. on Saturdays, October 5th, 12th, 19th, and 26th; November 2nd, 9th, 16th, 23rd, and 30th; December 7th and 14th, 1895. 2. The Town Hall, Caxton Street, Westminster, S.W., at seven p.m. on Sundays, October 6th, 13th, 20th, and 27th; November 3rd, 10th, 17th, and 24th; December 1st and 8th, 1895. 3. The Town Hall, Spa Road, Bermondsey, S.E., at eight p.m. on Saturdays, October 5th, 12th, 19th, and 26th; November 2nd and 9th, 1895. 4. Holloway Hall, Holloway Road, N., at eight p.m. on Saturdays, November 2nd, 9th, 16th, 23rd, and 30th; December 7th, 1895; February 1st, 8th, 15th, 22nd, and 29th; and March 7th, 1896.

Trained Nurses' Club, 12, Buckingham Street, Strand.—Lecture on Friday, October 25th, at a-quarter to eight, by Dr. Tom Robinson. Subject, "The Promises of Nature."

### Nurses in the Provinces.

TRAINING.

Whilst the progress made in metropolitan training schools is observed, commended, and encouraged, the position taken by some of the nursing staffs in provincial hospitals receives inadequate public acknowledgment. Perhaps, all things considered, this is for the ultimate advantage of the latter, as it gives them time to work on undisturbed towards perfection, and to show a bevy of well-trained probationers as the first signal to the outside world of two or three years' steady progress in the wards.

Much as the prevailing tone of a London training school depends on the matron, a provincial school relies even more on her for guidance. There is less variety in the life of a country hospital and many fewer visitors; the distractions being less the mutual ties of the nursing staff are stronger, and there is often a feeling of almost family life about a provincial hospital. The work is not of necessity lighter, and as a rule the hours on duty are quite as long, but there is more time to do things in because of the fewer outside interruptions.

With regard to certificates there seems little doubt of the value those given at large, well-managed provincial hospitals and General as well as Poor Law Infirmaries will prove to the nurses who have them.

Leeds.

The training school at Leeds Infirmary has a well-established reputation, and in many matters of detail has kept in advance of London institutions. A systematic course of training and the use of washing uniforms by all the members of the staff were common in Leeds when short training and stuff dresses were still in evidence in many metropolitan hospitals.

Bradford.

In planning the fine General Infirmary at Bradford it seems as if the nursing department had been altogether overlooked. The pleasant old wards and the fine new ones being admirable, but the lavatory arrangements of both (from the nursing point of view) leaving much to be desired in position and dimensions.

Again, from a nurse's point of view, the advantage of having an anæsthetising room adjoining the theatre is considerably detracted from by its entrance. The awkward descent by steps into the room being trying for the patient on whom the nurse's attention is centred.

The charge nurses are lodged in the infirmary, the probationers and night nurses in some unattractive old houses across the grounds. It is to be hoped that a great effort will be made later on to purchase these premises and build a nurses' home worthy of the infirmary and of the Bradford citizens. The present accommodation may surely be regarded as only temporary, and the matron has certainly done her best to brighten these very unpromising quarters. The whole place is kept beautifully clean, and the orderliness of the plain rooms puts to shame the condition of many more pretentious establishments. The sitting-room is pretty and homelike, and delightfully quiet, but there is no box-room, and only one bath-room for the probationers.

Probationers are admitted from twenty-one to twenty-five years of age, and the uniform is neat and pretty. The medals, recently reproduced in The Hospital, are exceedingly handsome, and worn with pride by the winners of these prizes.

The appointment of a night superintendent, which was made at the matron's suggestion, has been found to work well. Capes with hoods are provided for the nurses' wear in crossing the grounds in cold weather, and the matron, Mrs. Magill, may be congratulated on what she has already achieved for her staff.

Pianos and flowers in the wards show that the patients' pleasure is considered, but the red blankets covering the beds on a hot summer's day seem hardly consistent with the comfort of weak or feverish patients. In the beautiful children's ward the red blankets have a singularly inappropriate appearance in summer, and it is strange that no visitor has thought of replacing them with a set of coverlets more suited to the tropical heat this year.

Under the Poor Law.

Mill Road Infirmary, Liverpool, may be taken as a model one, being new enough to have all modern conveniences. The nurses' home is a fine detached building, containing a number of delightful bed-rooms on a larger scale than is usual, and they have wide windows and exceedingly pretty furniture. There are two sitting-rooms, comfortably fitted, and very good bath-rooms. The long corridors are floored with warm-coloured tiles, and there is a large recreation or lecture room. The night nurses have the top floor, and their rooms are quite cut off from the day ones and equally well fitted up. In the nurses' mess-room the meals are well served, and no pains are spared to make them as varied as is consistent with economical management. The neatly ordered tables and appetising-looking food show that due attention is paid to this important department.

Lectures are given to the probationers, and they appear to be thoroughly instructed, and, moreover, they do credit to their teachers by looking healthy, intelligent, and remarkably neat and nurse-like. The third year of training of the first batch of probationers is approaching completion, and many of them are doubtless looking forward with eagerness to the certificates which will be awarded in the new year.

The whole scheme of training is identical with that of a first-class hospital, and the condition of the wards and patients is most creditable. Remembering the short time that has elapsed since the opening of the new infirmary and the chaos out of which this exquisite order has been evoked, other matrons under the Poor Law may take courage. In making a tour of the wards it is pleasant to learn from charge nurses who came in when the first patients were transferred in scores from the old buildings, how quickly things settled down into order. The labour and the difficulties of getting such a huge establishment into working order seem forgotten in the present perfection of the place. The Guardians no doubt realise their good fortune in having secured in their matron a most efficient organiser as well as a thoroughly competent teacher and nurse. Courses of Lectures are given by Dr. Chaplin, the medical superintendent, who has been well to the fore in promoting trained-nursing at Liverpool.

At Bolton.

What Mill Road Infirmary is, Bolton Workhouse Infirmary must aspire to be. At present the two best departments are the isolation block, which is of modern construction, and the nurses' house, which is charming. It has a large garden and is just a pleasant roomy gentleman's house, which has been requisitioned for the recently-acquired nursing staff. The infirmary is old and inconvenient, deficient in what modern nurses consider absolute essentials for their work. But the cases are varied, and there is plenty of nursing work to be done and good instruction as to how best to do it.

The medical superintendent encourages Miss Hughes' schemes in every way, and the probationers who have settled down under certificated charge nurses to their three years' course have every prospect of finding plenty to learn. Miss Hughes is an enthusiastic teacher, as she showed when superintendent of the Bloomsbury Central Training Home for Queen's Nurses.

A new separate infirmary is urgently needed for this workhouse, but before that is provided it is evident that an efficient nurse training school will be firmly established at Bolton.

### The Ockley Scheme.

THE NAME OF NURSE.

We are indebted to Miss Broadwood for a copy of a pamphlet which we have read with interest, and now deal with for the benefit of those of our readers who have not already perused the scheme therein described.

"Nurses for Sick Country Folk" is the attractive title of an interesting record of work under "The Ockley System," compiled by Miss Broadwood and Miss Whitmore Jones. Naturally these pages also contain the usual arguments, by which creators of so-called "Cottage Nurses" attempt to justify the appropriation of the name and office of district nurses by women of very limited training. Could the word "Nurse" be altogether banished, and "Cottage Help" or "Mother's Help" substituted, the "system" which Miss Broadwood praises so eloquently would require no apologies, but then it might be loses one of its attractions. In recommending candidates for training Miss Broadwood says truly, that "no one has a right to benefit an individual at the expense of the community," a warning specially applicable in such a connection, and she draws attention to the need for health, sobriety, and honesty being associated with a real liking for the work of nursing. For district nurses in towns a high standard of training is allowed to be necessary, though few will agree with the writer that the market is already overstocked with the hospital lady nurse, or at any rate with those who are duly trained and certificated.

Pupil Nurses under Miss Broadwood's Scheme.

The ages of these vary from twenty-two to thirty-five, but even this limit may be set aside, it is stated, on occasions. Six months' preliminary experience in some district nursing institution, or the recommendation of persons to whom a woman has been well known for a year, makes an applicant eligible for entering as a pupil nurse. When accepted she must fulfil such duties as are assigned to her for three or six months in a ward or district. She receives training "such as is given to certificated monthly nurses," and is instructed in dressing wounds, bandaging, applying fermentations, poultices, leeches, the use of the clinical thermometer, enema apparatus and catheter, noting pulse, and various other nursing duties, besides invalid cookery, hygiene, observation and noting down of symptoms! Up to August of last year 190 women, presumably all of the cottager class, had gone through this form of training, and by this time the number doubtless has been considerably added to.

Training at Plaistow.

After repeated failures in a search for a hospital matron willing to consent to this shortened course of training for cottage nurses, Miss Broadwood's perseverance was rewarded by the discovery that Sister Katherine Twining would supply it at Plaistow. With this lady's great work in the extreme East of London most of our readers are acquainted, and Sister Katherine's reputation for success in training midwives and monthly nurses is second to none; whilst her devotion to the poor amongst whom she lives is remarkable.

Sister Katherine's System.

If Sister Katherine's system of training were available only for those whose previous experience in general hospital or infirmary wards required supplementing by systematic maternity and district nursing a great benefit would be conferred on the community at large, and she would provide for a long-felt want. "It is so difficult to secure district training," is such a common cry from nurses who wish to devote themselves to this particular branch. Herself a cultured woman, clever nurse, and midwife, Sister Katherine must be well aware of the drawbacks of short training which expediency has, nevertheless, caused her to countenance. Probably she would infinitely prefer pupils to remain with her one or two years, and the poor people to whom her life is devoted would assuredly greatly benefit by such a course. But a succession of pupils means a succession of fees and a supply of free nursing for the poor.

An Explanation.

Associations apply to Plaistow for such short terms of instruction for their future nurses as they fail to secure for them elsewhere, and charitable ladies are willing to pay the modest fee which, they think, will sufficiently equip a destitute widow for getting her daily bread "as a nurse." There is a preference for giving two women inadequate training rather than making one into a fully competent nurse. This offers a strong temptation to provide a series of attendants for the sick at Plaistow at a minimum cost, under the strict supervision of Sister Katherine and her permanent staff. As the responsibility rests with the latter, they doubtless make proper distinctions between the pupils' duties and their own; but after three or six months' training these village people leave, and bear the title in future of nurses. The after-responsibility of their work may be borne by rural committees, but must be shared to some extent by the establishments which supply them in such rapid succession.

A Question of Economy.

It is merely a question of money, but the manufacture of cheap imitations is inexpedient and false economy in the end. That these cottager women should be willing to combine the duties of nurses with those of charwomen, at the same rate of pay as the latter, shows that the superior title of a nurse has a certain commercial value. Not only is this woman of limited training called nurse under the Ockley scheme, but it is stated by Miss Broadwood (who, falling into the first person singular, causes us to overlook Miss Jones altogether) that "auxiliary or extra nurses" should be encouraged—*i.e.*, tidy and respectable women who can take up a convalescent case when the actual nursing is over.

An Unpractical Proposal.

It is also proposed in this pamphlet that matrons should encourage their nurses to instruct wardmaids in the art of nursing, and thus fit these young persons for becoming cottage nurses. It is even suggested they would take lower wages if this bait were offered. The injustice to hospital and infirmary of such a scheme appears to be quite overlooked, but wardmaids who have their time already fully occupied by the duties they are paid for doing must neglect these if they are to learn matters of actual nursing. Moreover the educated probationers, who are not like the wardmaids mere girls in their teens, might rightly resent the diversion of the instruction due to themselves into another direction. The duties of a matron include supervision of both nursing and domestic staffs, and it is her ambition to see that equal excellence is attained by each class of worker, which can only be done by a strict definition of the duties of each. With every confidence that the work of the Ockley Association (which includes a benefit fund in its scheme) reflects credit on its enthusiastic promoter, we must always protest against these excellent and valuable cottage helpers assuming a right to bear the title of nurse.

### A Private Hospital in Australia.

The private hospital opened in Melbourne in 1890 by Miss Marletri has proved an undeniable success, and its popularity has already necessitated the acquisition of larger premises than those originally taken. Two fine houses in Spring Street have been secured and altered into the Rokeby Private Hospital. The premises are new, but the permanent nursing staff consists of nurses who joined Miss Marlerti when she started her now well-known establishment. She trained in Melbourne, afterwards holding appointments at several hospitals, and to this experience has added a record of six hundred private patients, nursed since the opening of her home. A beautiful operating theatre forms an important part of the equipment of the new premises, and the sanitary arrangements are reported to be excellent.

### One Aspect of Tommy Atkins in Hospital.

(By One Who Has Nursed Him.)

When Tommy Atkins enters an Indian military hospital, or in his own language, "goes sick," he is escorted to hospital by a non-commissioned officer and handed over to the assistant surgeon on duty. Unless the patient's case is an urgent one, for the first twenty-four hours after his arrival he occupies a sort of intermediate state known as the "detained ward," where he stays until the following morning. Then he appears before the senior medical officer.

Tommy's ideas on the subject of medical officers are well defined, and he is not backward in expressing his views. For the habitual and successful shammer it is very important that on the occasion of a church parade or a route march (both of which functions have been known to exercise most pernicious effects on Mr. Atkins' health) the medical officer should be a good natured, easy-going person, who says benignly, "Very well, my man, a few days' rest in hospital will do you no harm."

Mr. Atkins salutes, departs to the ward, and sets down the medical officer as a "reel gentleman." On entering the ward, if there is a nursing sister in charge, he proceeds to make friends with her. His bed is allotted to him, and, after a bath, he is safely established in his new abode. All uniform is returned to the regiment (except helmet and boots), and patients wear hospital clothing of rather an artistic shade of blue. The garments are bright blue flannelette in summer, and thick pilot cloth coats of dark navy blue in the cold weather.

The first time one goes to a military hospital, after being accustomed to the spick and span wards of a large London hospital, the eye is struck at once by the untidiness of the collection of things arranged under the beds. The patient's boots, a tin basin, soap, blacking, brushes, &c., all are piled up, each in its own place, but the effect is very bad. All the patient's other possessions are arranged on an open teapoy, with three shelves, which stands beside each bed.

In each ward where there are severe cases, besides the nursing sister in charge, there is a staff of orderlies, who are each on duty eight to twelve hours, and a collection of native servants. With the natives Mr. Atkins wages a constant war. Many are the complaints of "them black niggers," which are poured into sister's ear. Were the whole of India searched for the most choice assortment of thieves and liars, the prize specimens would doubtless be found in the ranks of the Army Native Hospital Corps. To control them is difficult, if not impossible; for according to regulation, they may only be fined a certain very small sum each month. So that if a culprit commits a heinous crime on the first of the month, and is fined the maximum amount for it, he may behave as badly as he pleases, within certain limits, for the rest of that month, secure in the fact that his precious rupees cannot again be touched. For no other punishment, except that of a fine, does he care one single jot.

The nursing sister, besides being nurse, has to be amateur detective, policeman, and, if possible, thief catcher. But, in spite of all precautions, and of every possible check which white ingenuity can devise, the "black nigger" more often than not comes off victorious and effects a successful *coup*.

Tommy's amusements in hospital are few. He reads all the books and newspapers he can get hold of, and he is very fond of playing cards. One day the following little dialogue was overheard. An old soldier was teaching a "recruity" a new card game. The pupil was very stupid, so the teacher got disgusted, threw down the cards, and remarked in withering tones, "Lor! You h'are a stoopid. Ye're as stoopid as one them bloomin' young oificers!" At Christmas time some mild festivity is nearly always arranged for the men in hospital, with more or less success. Gratitude is not always gracefully expressed by Tommy, although no doubt it is felt by him. One year an ample tea was given to the men, followed by a "bran pie." The entertainment seemed to pass off well, but the only comment made next day to one of the hostesses was this: "That theer tea pawty o' yourn 'ave upset moy insoide!" A substantial breakfast given to a party of invalids on the morning of their departure for England elicited the remark, "Them eggs was too 'ard biled!"

So the time goes on until the patient thinks he is "fit for me dooty, sir," or the medical officer thinks Private Atkins has been in hospital quite long enough. So he is discharged, goes back once more to his khaki uniform and daily drills, perhaps with graceful adieux to the nursing sister, expressed thus: "Good-bye, Sister, they gives yer a very good name in barricks!"

### Notes from Melbourne.

(Communicated.)

The present is the time of annual meetings of hospitals and other charitable institutions. The Austin Hospital for Incurables is about to build suitable quarters for the nursing and domestic staffs. As it has been found that some inmates so improve in health as to cease to require either nursing or medical attention (when the cases become unsuitable to the nature of the hospital) a basis of agreement has been decided upon with a number of the metropolitan and country charitable institutions for an interchange of suitable patients.

A new wing—to be called the Aubrey Bowen Wing—is in course of erection at the Eye and Ear Hospital, and will be ready for occupation in December, the widow of the late Dr. Aubrey Bowen having given £3,500 for the purpose.

The enlargement of the infirmary department of the Women's Hospital is now nearing completion and should considerably decrease the number of deferred admissions. At present many women have to wait to come in for operations until they are almost past hope. At the annual meeting on August 8th four honorary medical officers were added to the staff of eight by the committee, because, as alleged, the present staff had been found insufficient to deal with the out-patients. The members of the staff stoutly opposed the increase, but the proposal of the committee was carried. There was much difference of opinion as to the division of duties, and the subject has to be further considered.

The Sydney Benevolent Asylum during this winter has been granting a maximum outdoor relief to mothers and children at the rate of 3s. per week. This being totally insufficient, a member of the board, Mr. J. Roseby, on August 14th, moved that the Government be asked to increase the allowance to 5s. The institution has been long established, and has a reserve fund of £20,000.

The closing of the doors of the City of Melbourne Bank will deprive several of the Melbourne hospitals for some time of good legacies, which they badly need, and might otherwise have touched at once, under the will of a retired mariner, who died in the Melbourne Hospital on August 8th. He left to the Melbourne, the Alfred, and the Homœopathic Hospitals, and to the Blind Asylum £150 each; to the Deaf and Dumb Asylum, £50; to the Women's Hospital, £10; and to the Salvation Army, £400. That a man with over £1,000 should choose to go to a hospital is significant of the high esteem in which he held such institutions.

Small-pox was again nearly introduced into the Colonies in July. A case occurred on board the *Lusitania*, and the case was landed at Colombo, but no notification of the fact was cabled to Australia, so that no preparations could be made at any of the quarantine stations. Three deaths from the disease occurred at the Adelaide quarantine amongst the passengers landed from the steamer.

Miss Isabella Rathie, matron of the Melbourne Hospital, was married on Thursday, July 18th, to Dr. W. H. Cutts. Miss Rathie was trained in the Edinburgh Infirmary, and was for some years matron of the Hobart Hospital (Tasmania) before being appointed to Melbourne. Dr. Cutts is a very old colonist.

### Everybody's Opinion.

[Correspondence on all subjects is invited, but we cannot in any way be responsible for the opinions expressed by our correspondents. No communications can be entertained if the name and address of the correspondent is not given, or unless one side of the paper only be written on.]

### HOURS OF ASYLUM ATTENDANTS.

"An Asylum Nurse" writes: In "News from the Nursing World," in a recent issue of The Hospital, I saw an article on the number of hours worked by hospital nurses. If our lunatic asylums may be classed as hospitals (mental) I should like to say a few words on the hours worked by nurses in these institutions. I must begin by mentioning the difference in the duties in general hospitals and in asylums. In the latter it is well known that in addition to being proficient in the art of nursing, an attendant has sundry things to contend with that a hospital nurse knows little of. If a patient in hospital insults or is violent to a nurse the law can punish that patient; if the same thing happens in an asylum the nurse is told that it is paid for by her wages. Nurses in hospital can sit down to take a little rest during hours off duty; not so with the asylum nurse, who has to be continually on her feet, if not in the wards, she may be struggling with refractory patients in the airing courts, watched by head attendants, deputies, and others, to see that she does not forget herself when being kicked or "wooled" by a patient, and with all this additional worry the asylum nurse works more hours than one in hospital and under more trying circumstances. She often takes her meals in the company of patients, many of objectionable habits, and who often grab at the nurses' food. Sixteen hours a day, or, say an average of ninety-six or a hundred a week, asylum nurses are on duty, and, in addition to having to earn a certificate, they must be able to take part in theatricals, sing, dance, &c. People may say this is recreation, but I think it is a form of it hardly appreciated by nurses who commence duty at six a.m., and do not get to bed till ten, eleven, or sometimes twelve o'clock. They are then worn out, often bruised about the body, and, like the "Village Blacksmith," have earned their night's repose.

### AN APPEAL FOR HELP.

Miss Mildred E. Staley, M.B.Lond., Physician-in-Charge, St. Stephen's Mission Hospital for Women and Children (S.P.G.), Delhi, Punjab, India, writes: Will you allow me to appeal through your columns for a nurse to assist in this hospital for women and children. There is a splendid work to be done in training native nurses and assistants, as well as in nursing many serious medical and surgical cases. The remuneration offered (Rs. 35 per month—about £2) is not sufficient to tempt any ordinary-trained nurse, with perhaps family claims upon her, to face the discomforts of the Indian climate. Should, however, there be anyone free from home ties who would be willing, for Christ's sake, to come and help us, she would receive a warm welcome from the two lady doctors in charge of this hospital. At present the whole burden falls on us; we have to see to the nursing, and do it ourselves in serious cases. Board and residence will be provided in addition to the salary.

### THE UNDERWORKED NURSE.

"A Nurse" writes from Paris: You suggest in last week's Hospital that "the Underworked Nurse" should employ part of her spare time in sewing. I wonder if she would make something for your Christmas competition if I sent her five shillings' worth of material? I have always wanted to make something myself, but have never had time. Personally, I find that with an "old and uninteresting case" I have just as much to do as I have for my present patient, who has a temperature ranging from 102 deg. to 105 deg. F. "Old and uninteresting" persons want so much companionship, reading aloud, writing their letters, and help even with their amusements. There are a hundred little things required that are not needed for a patient who is really ill. The Hospital last week was of special interest to nurses who work on the Continent, both as regards uniform and the Celsius Thermometre. The latter is not, as a rule, liked by English nurses; it is too "clumsy," and keeps the patient's chest so exposed during the ten minutes it has to remain in the axilla. You say patients do not go to fashionable hotels with their nurses for "pleasure," but I have had patients in all the principal hotels in Paris, and in each case the patient had been taken ill whilst stopping at the hotel, or else an accident has happened, and the patient must have a nurse. I never wear uniform in hotels, and am sure I can do my work just as well in a washing cotton blouse and apron, as with cap, collar, and cuffs. In private houses or apartments, of course, I always wear uniform. Last March I was nursing in an hotel, where there were nine other nurses at the same time. Fortunately, only two wore out-of-door uniform, or people would have thought the establishment was a nursing institution. I was in Cannes with a patient nearly two years ago, and always went to table d'hote. One night a nurse in uniform came in and was immediately asked to take a seat at a small table by the door and a screen was placed round her. Next day I met her in the hotel garden and spoke to her, saying I, too, was an English nurse, and felt sorry she had been made to feel uncomfortable at dinner the day before; and if she left off her cap and apron just during dinner, I told her no one would notice that she was a nurse, and she would be able to sit at the general table. She merely snubbed me in return for my advice, and told me that I seemed ashamed to wear uniform. She wondered how anyone could be a nurse and leave it off, for she considered the uniform one of the chief attractions of the nursing profession, and should always wear it. She did not put in an appearance at dinner that night, and I found she was placed at the couriers' table. Several people have told me that they have seen a screen put round a nurse in hotels both at lunch and dinner on the Continent and in London.

[Our correspondent's letter is a pleasant one, and shows the just estimate which she has formed of the claims of "old and uninteresting," as well as acute cases, on the services of the private nurse. Our correspondent evidently does her very best for those placed under her care. With regard to the long French thermometer we see no necessity for any exposure of a patient's chest if a little trouble be exercised in the arrangement of the blanket.—Ed. *T. H.*]

### "NURSES, THEIR UNIFORM, AND THE PUBLIC."

"One of Them" writes: Will you allow me to remark that, in your foot-note to the nurse who writes in last week's issue of your paper under the heading "Nurses, their Uniforms, and the Public" you overlook two very important facts. First, nurses as a rule only have a couple of hours "off duty" during the day, and possibly (not always) one day per month, and if twenty to thirty minutes of these precious two hours daily is to be spent in dressing and undressing, it does not leave them much time to spend with friends, especially if these live at some distance. Second, the average yearly vacation for nurses ranges from one fortnight to one month, so that if they are to appear in ordinary attire when on "recruit" or "off duty" they must either encumber themselves or their friends with a certain amount of wearing apparel which they only make use of for a very short period once a year, at the same time running the risk of providing food for moths. My own experience is, that there are very few nurses who can afford to keep such extensive wardrobes, although I am willing to admit that I think "private nurses" should "doff" their uniform when not actually at any case, for they as a rule have some few hours to spare to overhaul their apparel, which the hospital ones have not. I agree that always appearing in uniform does make our order conspicuous; but then, what are we to do?

## Figures and Tables

**Figure f1:**